# Reorienting Locus of Control in Individuals Who Have Offended Through Strengths-Based Interventions: Personal Agency and the Good Lives Model

**DOI:** 10.3389/fpsyg.2020.553240

**Published:** 2020-09-15

**Authors:** Nichola Tyler, Roxanne Heffernan, Clare-Ann Fortune

**Affiliations:** School of Psychology, Victoria University of Wellington, Wellington, New Zealand

**Keywords:** agency theory, Good Lives Model (GLM), locus of control, rehabilitation, offending

## Abstract

Having an external locus of control has been associated with a range of well-supported risk correlates of offending behavior. Further, individuals with an internal locus of control orientation are suggested to be more open to engaging in treatment and are also considered more likely to have successful treatment outcomes. In forensic settings, where individuals are subject to external controls and have little personal autonomy, it is important to consider what treatment approaches might be most successful in reorienting individuals’ locus of control. The Good Lives Model (GLM) proposes a strengths-based approach to the rehabilitation of individuals who have offended. Within the GLM, an external locus of control is suggested to be associated with a deficit in the primary good of agency. In this article, we will provide a brief overview of the literature on locus of control and its hypothesized role in offending behavior. We will discuss how an external locus of control orientation is related to personal agency and how strengths-based models, such as the GLM, may assist with reorienting locus of control in individuals who have offended through promoting personal agency.

## Introduction

Rotter (1966) defines locus of control as the degree to which a person perceives an outcome as being contingent on their own actions or those of external forces, existing along a continuum from a more internalized orientation to a more externalized orientation. Individuals who hold the belief that outcomes are dependent on their own behavior or personal characteristics are said to have an internal locus of control. In contrast, those with an external locus of control believe that life outcomes are determined by forces outside of their control (e.g., independent of their own actions or as a result of fate, luck, or chance), are dependent on powerful others, or are unpredictable due to the complex nature of the social environment.

The development of locus of control orientation is described within the context of Rotter’s (1954) social learning theory, where future outcome expectancies for specific or seemingly related events are strengthened through reinforcement. Individuals’ own experiences and reinforcement history are hypothesized to be related to the extent to which they attribute outcomes to their own actions (i.e., a more internalized or more externalized orientation). Thus, attitudes, beliefs, and expectancies associated with an individual’s locus of control orientation are suggested to develop, be reinforced, and strengthen through their interactions with others, the environment, as well as individual differences (e.g., cognitive development, feelings of alienation or powerlessness, need for autonomy or active mastery of the environment, and need for achievement; Rotter, 1966).

Rotter’s (1966) construct theory of locus of control has proved highly popular in the psychological literature, attracting much attention from researchers. However, while the construct of locus of control has garnered much attention over the years, it has been noted that over time “…the concept of locus of control or reinforcement may have become untethered from its original social learning theoretical roots” and that there has been a “proliferation of similar sounding terms” (Nowicki and Duke, 2017, p. 148). The different ways in which locus of control orientation has been operationalized within the forensic psychological literature provides one example of this.

Locus of control is of interest to forensic practitioners due to its relationship with well supported risk correlates for offending behavior (i.e., crime) and factors associated with treatment success (e.g., treatment readiness, engagement, and improvement on areas of need). However, within the forensic literature, locus of control has been operationalized in two, arguably distinct, ways. In some instances, locus of control has been operationalized similarly to Rotter’s (1966) definition, with an externalized locus of control relating to feelings of being a passive agent where events and consequences are perceived to be outside of the individual’s control, and an internalized locus of control related to feelings of empowerment and the ability to control or influence events and outcomes (e.g., McAnena et al., 2016). In contrast, other literature has defined or used locus of control as a proxy measure for accountability (i.e., the extent to which individuals take responsibility for their actions and behaviors; Beech and Fisher, 2002; Page and Scalora, 2004) and denial (Fisher and Beech, 1998). This second conceptualization is arguably more related to individuals’ *post hoc* reasoning about their offending and in this sense is vulnerable to cognitive processes such as justification or attempts to distance themselves from their misdeeds, as opposed to being a true reflection of their locus of control orientation generally. Therefore, it is somewhat misaligned with the social learning theory approach originally purported by Rotter, as it risks ignoring reinforcement experiences of individuals that may impact upon their beliefs relating to control and outcome expectancies, as well as their capacity for autonomy or self-direction.

Rotter (1966) suggests an individual’s motivation for autonomy or agency is likely to influence the strength of their outcome expectancies and therefore locus of control orientation. Personal agency refers to “an individual’s capacity for, and engagement in, intentional, goal-directed action” (Heffernan and Ward, 2017, p. 134) and the cognitive, affective, and individual learning experiences that influence this. Recent theoretical explanations of offending and rehabilitation have considered the role of autonomy and personal agency in human behavior (e.g., Heffernan and Ward, 2015, 2017; Thornton, 2016). Given the hypothesized relationship between locus of control orientation and agency, we suggest it may be helpful to consider how increasing an individual’s capacity for agency may also help to reorient an individual’s locus of control.

In this paper, we will first provide an overview of the hypothesized relationship between locus of control, offending behavior, and rehabilitation. We will then discuss how locus of control and personal agency are related and how it may be helpful to target the shared properties of these constructs (e.g., need for autonomy) in forensic treatment, as opposed to simply focusing on an individual’s ability to take responsibility for their actions. Finally, we will examine how strengths-based approaches to rehabilitation such as the Good Lives Model (GLM; Ward and Stewart, 2003) may help to reorient locus of control through increasing an individual’s personal agency. To ensure consistency with Rotter’s (1966) definition of locus of control, we have focused our review on empirical studies that have defined or measured locus of control using either Rotter’s (1966) Internal–External Control Scale or the Nowicki-Strickland Locus of Control Scales (Nowicki and Duke, 1974a,b), both of which were constructed to reflect Rotter’s original definition within his social learning theory (Nowicki and Duke, 2017).

## Locus of Control and Offending Behavior

Research has examined the association between locus of control orientation and offense-related variables including correlates of offending, treatment engagement and change, and recidivism. Individuals who have offended tend to report having a more externally oriented locus of control than non-offending individuals, with this finding replicated across both adult and adolescent samples and a range of behaviors, including sexual offending (e.g., Beck-Sander, 1995; Wood and Dunaway, 1997; Marsa et al., 2004), drunk driving (e.g., Cavaiola and Desordi, 2000), parents “at risk” of child abuse (e.g., Ellis and Milner, 1981), and shoplifting (e.g., Kelley, 1996). Further, within offending populations, individuals who have been involved in interpersonal violence (e.g., violence, sexual offending) have been found to have a more externalized locus of control compared to non-violent offending controls (Hollin and Wheeler, 1982; Marsa et al., 2004). Individuals with an external locus of control are also reported to be more likely to reoffend than those with an internal locus of control (Ollendick et al., 1980; Fisher et al., 1998; Halliday and Graham, 2000; Stevens et al., 2016; Tidefors et al., 2019). Thus, reorienting an individual’s locus of control to a more internalized direction appears to be an important target for rehabilitative programs.

### Correlates of Offending Behavior

Although individuals who have offended appear to be more likely to have a more externally oriented locus of control compared to non-offending individuals, this alone is unlikely to directly account for offending behavior. An external locus of control has been found to be associated with a range of well-supported risk factors for offending. More specifically, a more external locus of control has been associated with deficits in social competency (e.g., social skills, problem-solving ability, attachment style; Veneziano and Veneziano, 1988; D’Zurilla and Maydeu-Olivares, 1995; Marsa et al., 2004; Allan et al., 2007), coping (Carton and Nowicki, 1994; Gomez, 1997, 1998), self-esteem (Asberg and Renk, 2014), the presence of offense supportive attitudes (Allan et al., 2007; Chambers et al., 2008), and substance misuse (Cavaiola and Desordi, 2000).

Further, research from the general psychological literature suggests that an internal locus of control may function as a protective factor or psychological buffer (Page and Scalora, 2004). For example, several studies have reported that, compared to those with an externally oriented locus of control, individuals with a more internalized locus of control report significantly higher levels of self-esteem (Griffore et al., 1990), emotional and mental well-being (Armstrong and Boothroyd, 2007), adaptive coping (Kliewer and Sandler, 1992), and a more positive self-concept (Friedberg, 1982; Wood et al., 1996).

Limited research has examined the exact mechanism through which locus of control orientation influences psychological processes and physical behaviors, particularly in relation to offending. However, some researchers have hypothesized that an external locus of control may be linked to cognitive distortions (e.g., attributing blame to others for offending or viewing the self as uncontrollable; Chambers et al., 2008), increased impulsivity (e.g., lack of consequential thinking; Fisher et al., 1998), and may act as a defensive response to feelings of shame (McAnena et al., 2016). For example, perceived locus of control may shift toward a more external orientation following offending as the individual wishes to distance themselves from the behavior to retain a positive sense of self or avoid feelings of guilt. However, it is unclear whether cognitive distortions (e.g., justification, shifting blame, etc.) are actually reflective of locus of control orientation or whether they are an attempt to distance the self from certain behaviors (i.e., locus of control might generally be more internalized).

Research examining the relationship between locus of control and self-esteem suggests that, together, these factors may moderate psychological and behavioral outcomes. For example, Wallace et al. (2012) examined the relationship between self-esteem, locus of control, and self-reported aggression in a sample of 174 adolescents enrolled in a voluntary residential program. Wallace et al. (2012) found that higher levels of self-esteem were significantly associated with an internally oriented locus of control and that locus of control orientation moderated the relationship between self-esteem and self-reported aggression (both reactive and proactive). Further, Wallace et al. (2012) found that the combination of low self-esteem and an external locus of control was significantly related to higher levels of self-reported proactive aggression. In another study, Kliewer and Sandler (1992) examined locus of control and self-esteem as moderators between negative life events and psychological well-being in 238 young people. Kliewer and Sandler (1992) found that an internal locus of control orientation acted as a buffer to the effects of negative life events on psychological well-being. Further, the combination of an external locus of control and low self-esteem was associated with higher levels of psychological maladjustment.

## Locus of Control and Rehabilitation

### Treatment Readiness, Engagement, and Outcomes

A more internalized locus of control orientation has been linked with key factors associated with successful rehabilitation including treatment readiness (Chambers et al., 2008), amenability and engagement with treatment (Page and Scalora, 2004), and successful treatment outcomes (Fisher et al., 1998). Chambers et al. (2008) reviewed the literature on the influence of cognition on treatment readiness and engagement in rehabilitation for those who have committed violent offenses. They suggested that locus of control orientation is likely to influence individuals’ motivation and decision to change their behavior. More specifically, they argued that an external locus of control is likely to reduce a person’s commitment to behavior change and therefore their motivation and engagement in rehabilitative activities, as they are unlikely to perceive these as relevant or meaningful. Similarly, Page and Scalora (2004) reviewed the literature relating to locus of control, help-seeking, treatment participation, and treatment outcomes in young people who had offended and concluded that an individual’s locus of control orientation prior to treatment may provide an indication of their amenability to engage—with an external locus of control indicating a low level of treatment amenability. Empirical research supports these conclusions; for example, Groh and Goldenberg (1976) report an association between an internal locus of control orientation and engagement in prison-based occupational and educational programs.

While locus of control orientation is considered to be relatively stable (Rotter, 1966), research examining the effects of psychotherapeutic interventions on locus of control suggests that there is a dynamic quality to the construct, in that it is responsive to a range of interventions (i.e., it can be reoriented; Hunter, 1994; Newton, 1998; Page and Scalora, 2004; McAnena et al., 2016). In addition to being malleable to treatment itself, it has also been suggested that locus of control is associated with broader treatment success. For example, Fisher et al. (1998) examined the relationship between locus of control orientation and overall improvement on a range of outcome measures, administered pre–post treatment as part of a sexual offending treatment program. Fisher et al. (1998) reported that participants who were considered to have been “successfully treated” (i.e., had made a significant overall improvement on the battery of pre–post treatment measures) showed a significant shift toward a more internal locus of control pre–post treatment compared to those considered “unsuccessfully treated” (i.e., those who had not made a significant overall change). This finding suggests that rehabilitative activities that help to successfully reorient locus of control to a more internalized direction may also bring about broader improvements in other areas.

### Impact of the Criminal Justice System on Locus of Control Orientation

As noted above, locus of control orientation appears to be both responsive to intervention and important for bringing about change in other areas. However, forensic settings are arguably not the most conducive environments for supporting individuals to develop beliefs consistent with a more internalized locus of control. The very nature of imprisonment, detention, or community supervision restricts personal autonomy. For example, decisions about routine, recreational activities, sentence progression, and release back to the community are often outside of individuals’ control and dependent on powerful others. To illustrate, risk and parole board decisions rely on professionals’ opinions and input, and attendance at educational courses, offense-focused group rehabilitation, and work placements are reliant on the decisions of other people.

Further, time spent in prison is known to have a considerable impact on individuals’ psychological and emotional well-being. For example, longer stays in prison have been found to be associated with low self-esteem, depression, and reduced problem-solving abilities (Pugh, 1993), which, as noted above, are also associated with a more externalized locus of control. In addition, reduced outcome control has been associated with the development of “learned helplessness,” feelings of powerlessness (Goodstein et al., 1984), and weaker beliefs in free will (Rakos et al., 2008). Therefore, it has been argued that forensic environments may potentially promote and sustain an externally oriented locus of control by providing further reinforcing experiences (Kappes and Thompson, 1985). While impinging on agency is inherent within the practice of imprisonment, we are concerned that these restrictions may have the unintended consequence of reducing a person’s sense of personal agency in such a way that he or she may not perceive that change is possible (e.g., reinforce a more externalized locus of control), thereby undermining the goals of the criminal justice system. Given the apparent effects of forensic settings on both locus of control and agency, we will now consider the relationship between these two constructs.

## Locus of Control and Personal Agency

As outlined above, a number of overlapping and offense-related concepts associated with locus of control exist. Further, there are close conceptual relationships between locus of control and human capacities that have recently been theoretically linked with offending and rehabilitation; for example, self-control or self-regulation and concepts such as autonomy, motivation, free will or choice, and personal agency. Agency allows individuals to have control over their lives, to experience self-determination, and to feel as if they are acting without restriction or coercion. We suggest that locus of control orientation may reflect *an individual’s perception of their own capacity for agency* (e.g., “to what extent am I capable of engaging with the world to meet my various needs/goals?”), which in turn is supported by their perceptions of and expectations of their environment (Heffernan and Ward, 2017). This perception is informed by past experiences and other messages the individual has received about themself and their world (Rotter, 1966). For example, if a person experiences many opportunities to meet their needs/goals, others are supportive, they are competent/successful, and so on, they are likely to develop an internal locus of control and perceive that they are in control of their life and capable of dealing with situations that arise. On the other hand, where individuals experience the world as dangerous, full of others who wish to control them, lacking opportunities for them to meet their needs, and so on, they are likely to develop an external locus of control and feel powerless over their own life. This may result in an individual giving up and concluding that everything will always be this way (e.g., believing “there is no point trying to change as what happens to me is outside of my control, so I will only fail”) or developing new strategies in order to increase control over their life (e.g., creating illegal opportunities or attempting to control others). Due to its centrality within rehabilitation theories such as the GLM (Ward and Stewart, 2003) and recent theories of offending, we will now discuss personal agency and a number of agency-based theories that relate to locus of control. This includes the Predictive Agency Model (PAM; Heffernan and Ward, 2017), Thornton’s Theory of Dynamic Risk (Thornton, 2016), the Agency Filter Model (Serin et al., 2016), and theories of desistance (e.g., Sampson and Laub, 2005).

The PAM (Heffernan and Ward, 2017) was developed in response to perceived problems with the way explanations of offending tend to assume causality of dynamic risk factors or correlates of offending (e.g., antisocial attitudes, personality pattern). This model provides a general explanation of goal-directed behavior and as such is applicable to a range of behaviors (e.g., offending, substance use, desistance/change). The model asserts that behavior is guided by both mental representations held by individuals (informed by past experiences/learning) and emotion. These include the cognitive and emotional aspects of beliefs about the nature of the self, others, and world, as well as specific schemas or scripts relating to particular people or situations. These mental representations guide actions by operating like a template containing information, which is used by the individual to create situation-specific representations in “real time” and use these to make decisions. Decision-making is guided by the three types of expectation depicted in the Theory of Reasoned Action (TRA; Fishbein and Ajzen, 2010), those concerning perceived rewards, others’ reactions, and one’s own capability. In this model, locus of control would be conceptualized as a mental representation of one’s agency as more or less self-determined based on previous experiences. This impacts on decision-making (i.e., the TRA) because it influences whether or not the individual believes he or she is capable of acting to achieve a certain outcome.

Thornton (2016) (in his Theory of Dynamic Risk) suggests that behavior is motivated by a particular need or goal and that schemas are used as information to help the individual make a decision about how to act. These decisions are guided by expectations of one’s own abilities, likelihood of reward, and how others are likely to respond (i.e., the TRA; Fishbein and Ajzen, 2010). This theory was later applied to protective factors (those associated with desistance from crime) that are largely conceptualized as the inverse of risk domains; they are “families of related constructs” (Thornton et al., 2017, p. 30). There are four types of protective factor: internal, social support, professionally provided, and openness to professionally provided. Thus, this model sees protective factors as internal capacities (i.e., self-control, empathy, hope) and their manifestation within various arenas of life (Thornton et al., 2017), for example, influencing how open an individual is to engaging with professional support such as rehabilitation. Like the PAM, this model would conceptualize locus of control as a schema (or belief) influencing individuals’ perceptions of their capacity to bring about outcomes (or engage in successful agency), and it could be risky or protective depending on its orientation.

The Agency Filter Model (Serin et al., 2016) is another offense-focused theory that aims to explain the influence risk and protective factors have on an individual. This model suggests that individuals (at least to some extent) are able to choose how they react to external conditions (i.e., those that increase/decrease risk). These authors highlight the internal conditions that facilitate desistance from crime, and this includes hope, optimism, psychological flexibility, and self-efficacy (Serin et al., 2016). The “filter” that characterizes this model consists of an individual’s attitudes toward offending/desistance and the attributions they make about events that could lead to each (i.e., the meaning attributed to gaining employment, losing a relationship, etc.). This model would likely conceptualize locus of control as an important part of the “agency filter” containing beliefs about one’s own capacity for agency and attributions of outcomes/events as being internally or externally caused. For example, individuals with an internal locus of control who obtain employment might attribute this to their own hard work and efficacy and believe that, if they work hard, they will be able to progress and have a rewarding career, whereas individuals with an external locus of control might attribute this to luck and believe they are just as likely to lose this job through no fault of their own. These beliefs are likely to lead to different behaviors at work and will influence whether or not employment acts as a protective factor against offending for a particular individual. This highlights the importance of locus of control for the desistance process.

Desistance can be defined as the process of moving from active offending to reduced frequency/severity of offending, and eventually stopping offending altogether (Maruna, 2001). A number of mechanisms have been discussed as central to this process, both life events (e.g., employment, marriage) and personal shifts in identity and priorities (e.g., cognitive transformation and knifing off the past; Sampson and Laub, 2005). The internal changes associated with desistance are likely to be easier when one has an internal locus of control, for example, the transformation of one’s identity from “offender” to “non-offender” relies on one’s perceived ability to act differently in the face of external conditions (i.e., stressors and opportunities to offend). One cannot change their identity and behavior if they are not in control of the way they respond to external pressures and others’ expectations. Further, when encountering external support or events that often accompany the desistance process (e.g., employment or education opportunities, prosocial relationships and support, becoming a parent, etc.) an internal locus of control may facilitate prosocial responses.

Given the theoretical relationship between locus of control, agency, and offending, understanding and influencing an individual’s locus of control orientation are particularly important in forensic settings. The things that incarcerated individuals are able to control are limited, even choices about whether or not to make prosocial changes are influenced by the requirement that they engage with treatment in order to complete their sentence requirements and gain parole. Individuals who are incarcerated or on sentences in the community are expected to change, to become less criminal if they are to be released and/or trusted to reintegrate into society. It is not enough that an individual has served their time; they are also expected to demonstrate that they pose less risk to society than they did previously. Rehabilitation can be more or less coerced, ranging from mandated interventions, to those that involve various degrees of coercion (e.g., withholding privileges, denial of parole), to those that are completely voluntary (Parhar et al., 2008). More coercive rehabilitative activities have the potential to reinforce an external locus of control orientation. Thus, even when individuals have internal or intrinsic motivation to change, this motivation (and internal locus of control) may be undermined by outside pressures (e.g., coercion), known as the “undermining effect” (Ryan and Deci, 2000b). It is suggested that intrinsic motivation results in changes that last longer as they are not controlled by external conditions (Ryan and Deci, 2000a). In fact, research has found programs that are more coercive or mandated are less effective (in terms of reducing recidivism) than those that are voluntary, regardless of setting, suggesting that intrinsic motivation is more strongly associated with successful treatment outcomes and longer lasting change (Parhar et al., 2008).

Finally, even if individuals do decide change would be beneficial, if they do not feel they possess agency over their life, they are unlikely to feel motivated or competent enough to change. Because individuals undergoing forensic treatment are already likely to feel their autonomy is lacking, it is even more important that development of an internal locus of control is prioritized during preparation to make changes. With this in mind, the following section will explore interventions with individuals who have committed offenses that aim to shift their locus of control perception toward a more internal orientation, with a particular focus on strengths-based approaches (i.e., those that aim to increase individual’s skills and capacity to lead a meaningful and prosocial life).

## Reorienting Locus of Control Using Strengths-Based Approaches

Strengths-based approaches represent a relatively recent development in forensic psychological practice and arose in response to criticisms of more traditional rehabilitation models, such as the Risk-Need-Responsivity (RNR) model (Bonta and Andrews, 2017). The RNR model is a prevailing rehabilitation framework in the criminal justice system and contends that treatment is likely to be most effective when it is matched to the risk level, criminogenic needs, and personal characteristics of the individual (Bonta and Andrews, 2017). The RNR approach has amassed a body of empirical support; however, it has also been criticized as being “deficit oriented,” primarily concerned with risk management, external controls, and teaching individuals to avoid risky situations (Seligman and Peterson, 2003; Ward and Maruna, 2007; Ward et al., 2012). In comparison, strengths-based approaches encourage the building of strengths or resources necessary to create a personally meaningful and satisfying life, rather than just the avoidance of risk (Ward and Gannon, 2006). Strengths-based approaches are considered to be complementary to RNR (e.g., should be used alongside core principles). It is also suggested that additional attention to agency and self-efficacy may act to further decrease the risk of reoffending (Ward et al., 2007).

A number of strengths-based interventions for offending have been described in the literature, many of which are derived from the GLM (Marshall et al., 2017). While these interventions may vary in the extent to which they adhere to GLM principles, many incorporate proxies of a GLM approach (Willis and Ward, 2013; Marshall et al., 2017). Given this, in the following section, the GLM and its potential for promoting agency and a more internally oriented locus of control will be discussed.

## Strengths-Based Treatment: The Good Lives Model

The GLM (Ward and Stewart, 2003; Ward and Gannon, 2006) is a strengths-based rehabilitation model that encourages the building of strengths or resources necessary to build a good life, rather than just the avoidance of risk. The GLM is based on the assumption that offending, like all human behavior, is motivated by primary human goods (valued states or ways of being, such as happiness, inner peace, mastery, relatedness, autonomy, creativity, etc.). Persons have different ways of meeting these needs based on their own personal capacities and the external resources or opportunities available to them. For example, one might meet their need for autonomy through having an academic career where they are free to choose what they wish to research, while another may exercise autonomy through moving out of their parents’ home or leaving a controlling relationship. It is problems with attaining these goods that lead to harmful or offending behavior, for example, problems with the means used (i.e., they harm others) or conflict between different needs. For instance, if a person feels unable to meet his or her need for autonomy generally, he or she may engage in self-destructive behaviors to regain some sense of control (e.g., restrictive eating, self-harm, substance abuse) or he or she may attempt to control others through the use of violence, coercion, or intimidation. In overly restrictive contexts, such as prison, an individual may have very limited means available to meet a range of needs, including autonomy.

It has been suggested that one of the aims of antisocial/criminal behavior can be to demonstrate personal agency and control when individuals perceive they lack this in other aspects of their lives (Maruna, 2001), making this an important part of rehabilitation. Locus of control orientation is often not directly addressed as part of interventions for offending (e.g., through a specific module or dedicated sessions), and this is also true of GLM-informed interventions; instead, a whole program approach is proposed to support individuals to develop a more internally oriented locus of control through promoting agency and building skills. This extends from the underlying aims and ethos of the treatment program to the assessment process and the intervention content and delivery (Willis et al., 2012). In the following sections, we will discuss the mechanisms through which GLM-informed interventions promote personal agency and reorient locus of control. We will also highlight how strengths-based approaches like the GLM may prove helpful in forensic contexts, where opportunities to exert autonomy are restricted and experiences reinforcing an external locus of control are likely to be prevalent (see Table 1 for a summary).

**TABLE 1:** Impact of forensic environment on agency and locus of control and application of risk reduction vs. strengths-based approaches.

Features of forensic environment	Impact on agency and locus of control	Risk reduction interventions	Strengths-based interventions
Coerced rehabilitative activity (e.g., expected to engage to gain parole).	Undermine motivation to change through lack of agency.	Assumes capacity for agency already exists.	Collaborative treatment planning and goal setting. Use GLM to identify areas of treatment need. Positive therapist features (e.g., genuine, warm, unconditional positive self-regard, challenging). Develop capacity for agency through marking progress toward personal goals. Transparent communication.
Lack of control over routine, associates, food choices, etc.	Restrict personal choice. Increase feelings of disempowerment, loss of control.	May reinforce restrictions through setting avoidance goals and focus on external constraints.	Setting approach goals rather than avoidant goals. Having a flexible approach to treatment. Collaborative decision-making.
Incapacitation–restriction of movement and removal from society.	Loss of freedom. Limited control over external environment.	May reinforce incapacitation through “risk removal” approach.	Adopting a holistic approach to treatment—focusing on areas relevant to reducing risk as well as those that are important to the individual. Setting and working toward personally meaningful goals including those that can be achieved within the secure environment and steps that can be taken toward goals within this setting.

### Good Lives Model and Treatment Goals

The GLM is grounded in the ethical concepts of universal human rights and human dignity (Ward and Syversen, 2009). This is seen in the strong emphasis placed on human agency (Purvis et al., 2011; Willis et al., 2012). In GLM-informed rehabilitation programs, this focus on agency is seen throughout both the assessment and treatment process through the emphasis placed on supporting individuals to identify personally meaningful goals, working collaboratively to develop a plan for achieving these goals in a prosocial manner and giving individuals the space to implement their life plans (Willis et al., 2012).

The treatment goals of GLM-consistent interventions are also aligned with promoting agency. Historically, offense-focused rehabilitation efforts have been primarily focused on the management of risk and with that has come a focus on avoidance goals (Fortune et al., 2012). This means that the focus has been on individuals *avoiding* specific high-risk states and situations. For example, a goal for someone who has sexually offended against children might be to avoid places where children congregate, such as schools and parks, or avoiding feeling lonely, which may increase his or her risk of using the internet to access child exploitation materials. It has been argued that this focus on avoidance goals negatively impacts upon the engagement and motivation of individuals (Fortune et al., 2012; Fortune, 2018). In contrast, strengths-based rehabilitation approaches focus on approach goals, those that move an individual toward valued outcomes. For example, rather than attempting to avoid children or feelings of loneliness, an individual might have the goal to develop close friendships with peers (i.e., building a support system and social skills). In both cases, risk may be reduced, but in the approach goal scenario, the individual is focused on moving toward a valued outcome (i.e., relatedness) rather than avoiding risk. In GLM-informed rehabilitation programs, this means that the emphasis is on supporting individuals to identify approach goals that are personally meaningful and developing the capacity and competency to achieve these. The goal of therapy is to equip individuals with the skills, knowledge, resources, and supports necessary for them to realize their goals in socially acceptable ways, without causing further harm to themselves or others. Treatment also supports individuals to ensure balance in their life plan and that they reduce any conflict that might exist between their various life goals.

The GLM’s collaborative approach to identifying personally meaningful goals and developing an individualized plan to achieve these is one key strategy for promoting agency and empowering individuals. Within the pretreatment assessment, emphasis is placed on identifying what individuals hold most important in life through identifying the primary goods that are prioritized by them, how they have gone about attaining these previously, and identifying problems or obstacles with achieving these (Willis et al., 2012; Barnao, 2013). From this, a personalized good lives rehabilitation plan can be developed, outlining the individual’s goals and the steps needed (both in prison and upon release) to achieve these (for an example of good lives goal planning, see Barnao, 2013), which in turn informs case formulation and treatment planning (Willis et al., 2012). Adopting a collaborative person-centered approach from the outset helps to promote client agency and encourage the development of a more internalized locus of control, empowering individuals by enabling them to have input into their own treatment when in an environment within which they have little autonomy. Further, following assessment, clients are informed that the aim of treatment (or the treatment plan) is to support them with developing the skills and resources needed to achieve their goals and lead a life that is personally meaningful, while also reducing their likelihood of reoffending (i.e., to develop the means to have control over life outcomes through prosocial goal seeking; Willis et al., 2012).

### Good Lives Model Treatment Content and Delivery

The GLM approach has been found to have a positive impact on treatment motivation and engagement (Mann et al., 2004; Gannon et al., 2011), something which has been found to be low in individuals with a more externalized locus of control (Page and Scalora, 2004; Chambers et al., 2008). Further, it has been suggested that building motivation increases belief in justice-involved individuals that their goals are attainable (i.e., that they are achievable and that they have control over how they attain these; Fortune, 2018).

As noted earlier, GLM-consistent treatment programs promote agency not only through their aims, orientation, and pre-assessment but also through the way in which content is presented and delivered. An important component of GLM treatment is to support individuals to develop their understanding about how best to achieve their goals (Fortune, 2018). A GLM approach to treatment would seek to support individuals to develop skills to increase capacity for agency (e.g., understanding and managing emotions, problem-solving) and identifying and supporting engagement with opportunities that result in mastery and a sense of agency being achieved without using harmful actions (external capacities; i.e., education, leisure, or work programs in areas of existing interest, skill, knowledge, or ability) (Langlands et al., 2009; Fortune et al., 2015).

For example, McAnena et al. (2016) explored the role of locus of control and its relationship to treatment outcomes in a sample of 185 males who were referred to a non-GLM community treatment program for sexual offending (The Challenge Project). A more externalized locus of control was significantly positively correlated with risk scores on the STATIC-99 risk assessment measure (Hanson and Thornton, 2000). Further, locus of control was found to significantly shift from a more external to a more internal orientation post treatment. From this, McAnena et al. (2016) concluded that locus of control represents a potentially meaningful measure of treatment change. Subsequently, The Challenge Project treatment program was revised to include a greater emphasis on empowerment (agency) through increased collaboration, including providing clients with training on completing their own evidence-based risk assessments (Craissati, 2018).

Another core tenet of the GLM approach that promotes agency is the development of a strong therapeutic alliance. Therapist characteristics are known to play an important role in developing therapeutic rapport. However, the use of constructive and collaborative approaches when delivering treatment has also been reported to evoke intrinsic motivation and autonomy (Marshall and Burton, 2010). Involving individuals who have offended in formulating their offending and treatment needs and identifying ways in which they can achieve their life goals, while simultaneously addressing factors that may act as obstacles to attaining these (i.e., criminogenic needs), can provide individuals with a sense of hope and belief that they can control their life outcomes (i.e., a more internalized locus of control).

The research discussed in this section highlights some of the key ways in which strength-based approaches promote agency and through which they can reorient an individual’s locus of control. Through focusing on personally meaningful life goals and adopting a collaborative approach to treatment, GLM-consistent interventions can create opportunities for individuals to develop a more internally oriented locus of control in a restricted environment. In the next section, we will describe studies that have evaluated the efficacy of GLM-informed interventions in reorienting locus of control to examine the effectiveness of these.

### Effectiveness of Good Lives Model Approaches in Reorienting Locus of Control

While the GLM is used as a rehabilitative framework internationally, empirical evaluations of GLM-informed/consistent interventions are still in their infancy, with a lack of evaluation of post treatment reoffending (Willis and Ward, 2013; Marshall et al., 2017). That said, a small number of within-treatment evaluations have examined the effectiveness of GLM interventions with locus of control as an outcome measure, as well as an emerging body of research examining the contribution of the different components of the GLM to the desistance process. These studies and their findings will be briefly described here (see Table 2 for an overview).

**TABLE 2:** Summary of studies examining outcomes for GLM-informed interventions on locus of control orientation.

Author	Intervention	Sample	Measure of locus of control	Outcome
Barnett et al. (2014)	CSOG and TVSOG compared Relapse Prevention version to GLM version	Adult males with a conviction for sexual offending. CSOG (RP) = 163 TVSOG (RP) = 158 CSOG (GLM) = 105 TVSOG (GLM) = 97	Nowicki–Strickland Locus of Control Scale (Nowicki, 1976)	GLM completers showed a greater shift toward a more internalized locus of control post treatment completed to RP completers. A larger proportion of GLM completers showed a “functional score” on locus of control post treatment than RP completers.
Gannon et al. (2011)	Good Lives Sexual Offender Treatment Group (SOTG) for men with a mental illness	Adult males with a history of sexual offending Treatment = 5	Nowicki–Strickland Locus of Control Scale (Nowicki, 1976)	Locus of control scores within normative range pre treatment. 3/4 participants showed a small shift toward a more internalized locus of control post treatment.
Gannon et al. (2015)	Firesetting Intervention Program for Prisoners (FIPP)	Adult men and women with a history of firesetting Treatment = 55 Comparison = 45	Nowicki–Strickland Locus of Control Scale (Nowicki, 1976)	40.7% of FIPP participants showed a notable change in their locus of control orientation compared to 33.3% of the comparison group (*p* = 0.06).
Harkins et al. (2012)	N-SOGP compared Relapse Prevention (RP) version to Better Lives (GLM) version	Adult males with a conviction for sexual offending RP = 701 GLM = 76	Nowicki–Strickland Locus of Control Scale (Nowicki, 1976)	66% of the GLM participants demonstrated clinical change pre–post treatment on socio-affective measures (including locus of control) compared to 60% of RP participants.
Middleton et al. (2009)	iSOTP	Adult males convicted of an internet based sexual offense Treatment = 264	Nowicki–Strickland Locus of Control Scale (Nowicki, 1976)	Statistically significant shift to a more internalized locus of control observed post treatment.
Tyler et al. (2018)	Firesetting Intervention Program for Mentally Disordered Offenders (FIP-MO)	Adult men and women with a history of firesetting Treatment = 52 Comparison = 40	Nowicki–Strickland Locus of Control Scale (Nowicki, 1976)	Participants in the treatment group reported a slightly more externalized locus of control post treatment relative to the comparison group.

*RP, Relapse prevention; GLM, Good Lives Model.*

In a United Kingdom sample of males convicted of sexual offending, Barnett et al. (2014) found that individuals who completed a GLM-consistent program (*n* = 202) were more likely to display a “treated profile” post treatment than those in the Relapse Prevention program (*n* = 321). The determination of a “treated profile” included consideration of scores on five psychometric measures including the Nowicki–Strickland Locus of Control Scale (Nowicki, 1976). In a study of a program for individuals convicted of an internet-related (sexual) offense (i-SOTP) in England and Wales, Middleton et al. (2009) also found a positive relationship between GLM-consistent treatment and locus of control. Participants (*n* = 264) completed a battery of psychometrics including the Nowicki–Strickland Locus of Control Scale (Nowicki, 1976), with a significant change noted on this scale between pre and post treatment. This change was also noted in other areas of socio-affective functioning (e.g., self-esteem, emotional loneliness, cognitive and motor impulsivity) and pro-offending attitudes (victim empathy and cognitive distortions).

In another example, Gannon et al. (2011) reported preliminary findings from an evaluation of a Good Lives Sexual Offending Treatment Group (SOTG) in the United Kingdom, designed for males experiencing mental health difficulties. Using case study descriptions (*n* = 5), Gannon et al. (2011) report that, despite their differential and complex needs, participants made progress during the program on some key indicators, including developing a more internalized locus of control. Although all individuals had locus of control scores within the normal range pre treatment, in four out of the five case descriptions, issues related to autonomy were associated with their offending, and in one case description, the individual clearly identified an external locus of control, describing himself as having “little control” over his index offense (p. 163).

In another study, Gannon et al. (2015) evaluated the effectiveness of a GLM-informed treatment program developed for adult males in prison with a history of deliberate firesetting: *The Firesetting Intervention Program for Prisoners* (FIPP; Gannon, 2012). A battery of psychometric assessments was completed pre and post treatment and at 3-month follow-up, which assessed a range of factors associated with deliberate firesetting, including locus of control orientation. Treatment completers’ (*n* = 54) scores on the psychometric measures were compared with those of a comparison group (*n* = 45) who resided at prisons where the FIPP treatment was not available. Gannon et al. (2015) found that 40.7% of FIPP participants showed a notable change in their locus of control orientation (assessed using individual effect sizes) compared to 33.3% of the comparison group. This effect represented a non-significant trend in favor of the treatment group (*p* = 0.06) and was maintained at follow-up for FIPP participants but not for the comparison group.

In addition to treatment evaluation studies, there has also been some qualitative research exploring agency, the GLM, and the process of desisting from further offending. Barnao et al. (2015) examined 20 forensic patients’ perceptions of rehabilitation using thematic analysis. Agency was identified as one of seven key themes that characterized participants’ experiences of the forensic rehabilitative context. More specifically, “a lack of control overshadowed participants’ experience of compulsory detention” (p. 1036). Following this, Barnao et al. (2016) explored the impact of a brief GLM intervention using comparative thematic analysis of pre post treatment interviews with five forensic patients. Loss of agency, disempowerment, and feelings of being controlled were reported as key features of participants’ lives on the forensic mental health ward pre treatment. However, an increased sense of agency was associated with perceived change post treatment in three out of five participants. Features of GLM treatment such as shared decision-making, transparent communication, progress toward personal goals, and self-determination (e.g., feeling capable of doing positive things for oneself) were identified as contributing toward an increased sense of agency.

Wainwright and Nee (2014) looked at offending and the process of desistance in the Preventing Youth Offending Project (PYOP), a community-based (non-residential) GLM consistent program in the United Kingdom. Interpretative Phenomenological Analysis of semi-structured interviews with seven individuals aged 10–18 years who all started their criminal behavior prior to adolescence, identified four key themes social awareness, self-development, self-hope, and self-identity, with self-identity “assuming both a salient and influencing position over the other three” (p. 172). Within these themes, the importance of autonomy, along with relatedness, and competence, were highlighted. In terms of autonomy, those desisting from offending had a belief in their own ability to control their behavior and saw this as a personal choice, rather than being imposed on them by others. For this group, it became clear that developing an internal locus of control was a critical part of the desistance process (Wainwright and Nee, 2014).

It is also important to note that there has been some research that has found little or no association between GLM-informed rehabilitation and changes in locus of control orientation. For example, Harkins et al. (2012) compared the effectiveness of a GLM-informed sexual offending treatment program (*n* = 76) with that of a traditional relapse prevention approach (*n* = 701). Treatment effectiveness was measured by comparing attrition rates and change on participants’ pre–post treatment scores across a battery of psychometrics including the Nowicki–Strickland Locus of Control Scale. Harkins et al. (2012) found no difference in the attrition rates between the two treatment modalities. No measure-specific outcomes were reported for locus of control orientation pre–post treatment, however, a slightly higher proportion of participants in the GLM condition reported an improvement on the domain of socio-affective functioning (which included locus of control) compared to those in the relapse prevention condition (66% vs. 60%); however, this difference was not significant. In another study, Tyler et al. (2018) evaluated the effectiveness of a GLM-informed intervention for adults with a history of firesetting and a mental health diagnosis by comparing pre–post treatment psychometric scores on a range of treatment targets (including locus of control) for treatment completers (*n* = 52) with those of a treatment-as-usual comparison group (*n* = 40). Tyler et al. (2018) found that participants in the treatment group showed a larger pre–post treatment shift toward a more externalized locus of control than the comparison group; however, this difference was not statistically significant.

## Summary and Concluding Comments

Justice-involved individuals often present with an externally oriented locus of control that has been associated with a range of issues including well-known correlates of offending, lower levels of motivation and engagement with treatment, and poorer treatment outcomes (i.e., increased risk of offending). Locus of control is related to the human good of agency or autonomy and is viewed as one of the needs that all individuals prioritize. Justice-involved individuals often lack the relevant internal and external capacities to achieve personal agency. For individuals with low levels of personal agency, or an externally oriented locus of control, strengths-based interventions such as the GLM promote the development of agency using a whole-program approach (from their aims and orientation through assessment and treatment) while also supporting individuals to develop the skills to continue to live a personally fulfilling life. Early research evaluating the effectiveness of GLM-consistent programs suggests that these have the potential to positively reorient agency and locus of control within forensic environments. Given forensic settings may provide experiences that further reinforce an external locus of control, GLM-informed interventions represent a potentially promising way to overcome these barriers and to support locus of control reorientation within this environment. However, despite promising early findings, research evaluating the effectiveness of strengths-based interventions, and more specifically their ability to reorient locus of control, is still very much in its infancy. Therefore, further research is needed before any definitive conclusions can be drawn about the extent to which such approaches may promote personal agency and an internal locus of control above that of traditional risk avoidant approaches.

## Author Contributions

All authors listed have made a substantial, direct and intellectual contribution to the work, and approved it for publication.

## Conflict of Interest

The authors declare that the research was conducted in the absence of any commercial or financial relationships that could be construed as a potential conflict of interest.
